# The complete chloroplast genome of *Calyptothecium philippinense* Broth. (Pterobryaceae, Hypnales)

**DOI:** 10.1080/23802359.2024.2412232

**Published:** 2024-10-07

**Authors:** Haifeng Luo, Yu Miao, Wang Lu, Nanqiang Li, Ningning Yu, Yin Li, Wei Han

**Affiliations:** a College of Chemistry and Materials Science, Fujian Normal University, Fuzhou, China; b College of Life Science, Hebei Normal University, Shijiazhuang, China; c School of Resources and Chemical Engineering, Sanming University, Sanming, China; d State Key Laboratory of Systematic and Evolutionary Botany, Institute of Botany, Chinese Academy of Sciences, Beijing, China

**Keywords:** *Calyptothecium philippinense*, Pterobryaceae, chloroplast genome, phylogenetic analysis

## Abstract

The genus *Calyptothecium*, currently comprising ca. 30 species worldwide, is the largest genus within the family Pterobryaceae. However, a comprehensive taxonomic revision of this genus is lacking. *Calyptothecium philippinense* Broth. 1899, a moss species widely found in the tropical regions of Asia, is characterized by the unique rugose leaves and large auriculate leaf bases. In this study, we sequenced the complete chloroplast genome (CPG) of *C. philippinense* using the Illumina NovaSeq 6000 platform. The length of the CPG of *C. philippinense* was determined to be 124,513 bp, with an AT content of 74%. The CPG of *C. philippinense* exhibited a standard quadripartite structure, consisting of one small single-copy (SSC) region (18,541 bp), one large single-copy region (LSC) (87,222 bp), and two inverted repeat (IR) regions (9375 bp each). A total of 126 genes from the CPG of *C. philippinense* were annotated, including 82 protein-coding genes, eight ribosomal RNA genes, and 36 transfer RNA genes. Phylogenetic analysis based on the CPGs of 25 bryophyte taxa revealed that the three Pterobryaceae species *C. philippinense*, *Calyptothecium hookeri* (Mitt.) Broth. and *Pterobryopsis orientalis* (Müll. Hal.) M. Fleisch. formed a robust clade. The findings could facilitate more accurate classification and help elucidate evolutionary relationships within *Calyptothecium*.

## 1. Introduction

The genus *Calyptothecium*, initially established by Mitten in 1868, has since grown to encompass approximately 30 species (Frey and Stech 2009), making it the largest genus in the family Pterobryaceae. These species exhibit a wide geographical distribution, primarily found in the pantropical and Southeast Asia regions. However, there is substantial disagreement among bryologists regarding the classification of some species within this genus, owing to a dearth of molecular data. For example, *Calyptothecium tumidum* (Dicks. ex Hook.) M. Fleisch. was regarded as an identical species to *Calyptothecium philippinense* Broth. (Dixon 1937; Bartram 1939; Gangulee 1976; Hyvönen 1989). Noguchi (1985) demoted *Calyptothecium japonicum* to a synonymous status with *C. philippinense*. Similarly, *Calyptothecium bernieri* Broth. was recognized as a synonym for *Calyptothecium urvilleanum* (Müll. Hal.) Broth. (Noguchi 1985) or *Calyptothecium recurvulum* (Müll. Hal. ex Broth.) Broth. (Hyvönen 1989; Tan and Iwatsuki 1991). Based on morphological evidence, Yu and Jia (2015) treated *C. bernieri*, *C. japonicum* and *C. tumidum* as synonyms of *C. philippinense*. What’s more, at present, only two CPGs have been published in Pterobryaceae (Han et al. 2022a, 2022b). *C. philippinense*, a moss species distinguished by its uniquely rugose leaves and large auriculate leaf bases, was native to various countries across Asia and the Pacific, such as China, Japan, India, Sri Lanka, Burma, Indonesia, the Philippines, Papua New Guinea, New Caledonia, and Fiji (Yu and Jia 2015). In this study, we assembled and characterized the CPG of *C. philippinense*, providing molecular evidence for further taxonomic revision and phylogenetic studies of *Calyptothecium*.

## 2. Materials and methods

### 2.1. Sampling

**Figure 1.**
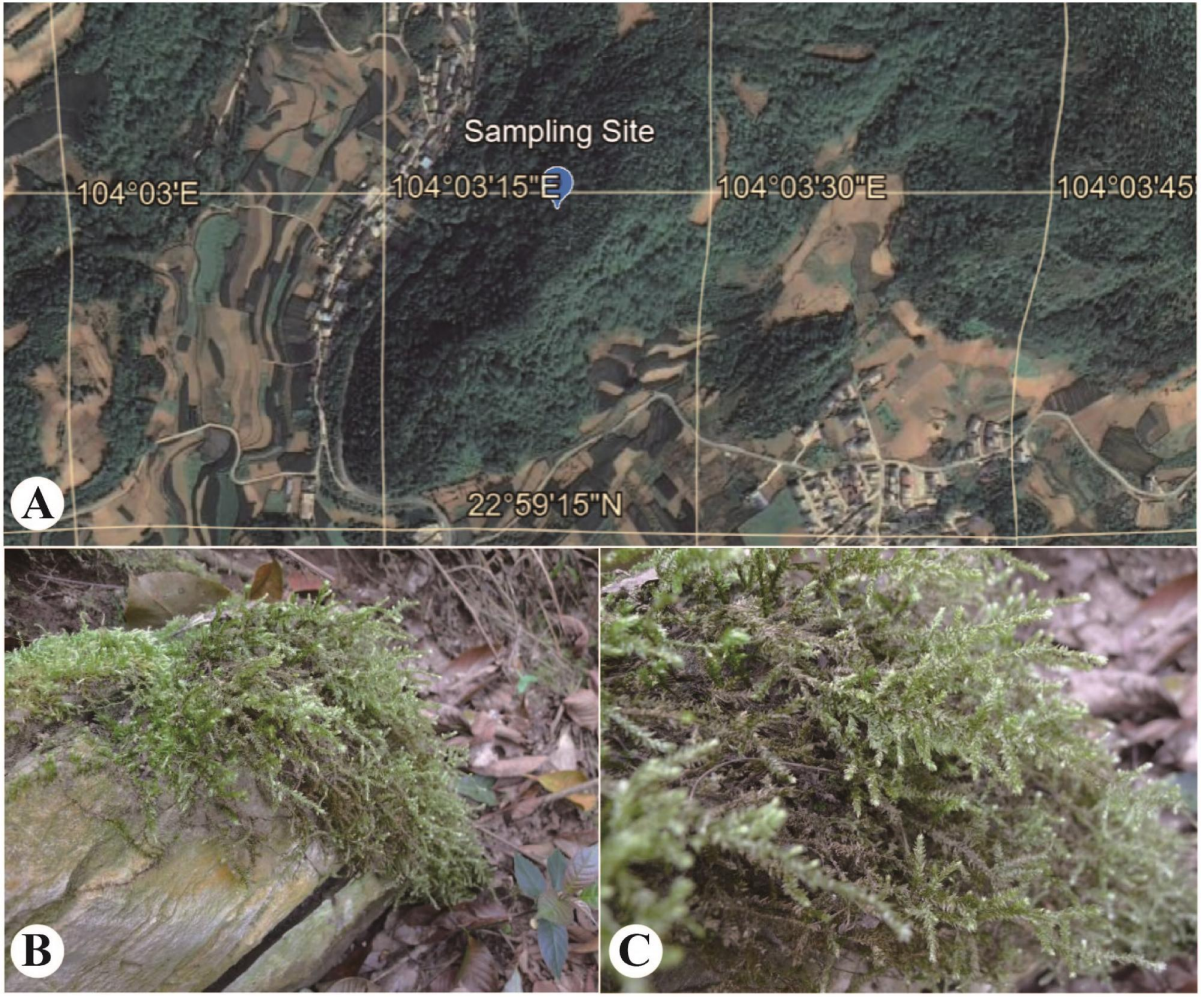
The picture of the collected *C. philippinense* sample. This image shows the sampling position (A), habitat (B), and photo of *C. philippinense* in the sampling site (C), which were recorded by Wei Han.

The plant material of *C. philippinense* was collected from Yaoshang village (22.9915° N, 104.05644° E; altitude. 1754 m), Maguan County, Yunnan province, China (Figure 1), and was identified by Dr. Ningning Yu. The voucher specimen (Wei Han HW346) was deposited in National Herbarium (PE, Ningning Yu, yuning@ibcas.ac.cn), Institute of Botany, the Chinese Academy of Sciences (IBCAS) under the barcode numberPE02163216.

### 2.2. DNA extraction and NGS sequencing

First, a DNAsecure Plant Kit (DP320 TIANGEN Biotech, Beijing, China) was used to extract total genomic DNA following its manufacturer’s instructions. Genomic DNA was then broken into fragments of an average length of 350 bp using Covaris (M220 Focused-ultrasonicator). Next, the NEBNext Ultra II DNA Kit was used to construct the DNA library for next generation sequencing (NGS). Finally, the DNA library was sequenced on the Illumina NovaSeq 6000 sequencing platform, and the raw paired-end reads were assembled into contigs using the software GetOrganelle (Jin et al. 2020).

### 2.3. Assembly and annotation

GetOrganelle (Jin et al. 2020) software was used to assemble the CPG based on the clean reads. The chloroplast genome was then automatically annotated using GeSeq (Tillich et al. 2017) and CPGAVAS2 (Shi et al. 2019). According to the results of automatic annotation, the manual adjustment was made in Geneious v9.0.2 (Kearse et al. 2012) with the reference of *Calyptothecium hookeri* (OL405127). Finally, A circular map of the CPG of *C. philippinense* and cis- and trans-spliced genes was generated using CPGview (Liu et al. 2023) and PMGmap (Zhang et al. 2024).

### 2.4. Phylogenetic analysis

To investigate the phylogenetic position of *C. philippinense*, we downloaded the CPGs of this species and 24 other representative species from the order Hypnales from the NCBI database, and *Myuroclada maximowiczii* and *Barbella flagellifera* were chosen as outgroups for the analysis. These sequences were aligned using MAFFT v7. TrimalV1.4 was used to cut the poorly compared sites (Capella-Gutiérrez et al. 2009). Finally, Bioedit v7.0.8.0 (Hall 1999) was used to manually check the results. A maximum likelihood (ML) phylogenetic tree was constructed using GTR+F + I + I + R3 model (tested using ModelFinder) in PhyloSuite v.1.2.3 (Ronquist et al. 2012; Nguyen et al. 2015; Kalyaanamoorthy et al. 2017; Zhang et al. 2020).

## 3. Results

**Figure 2.**
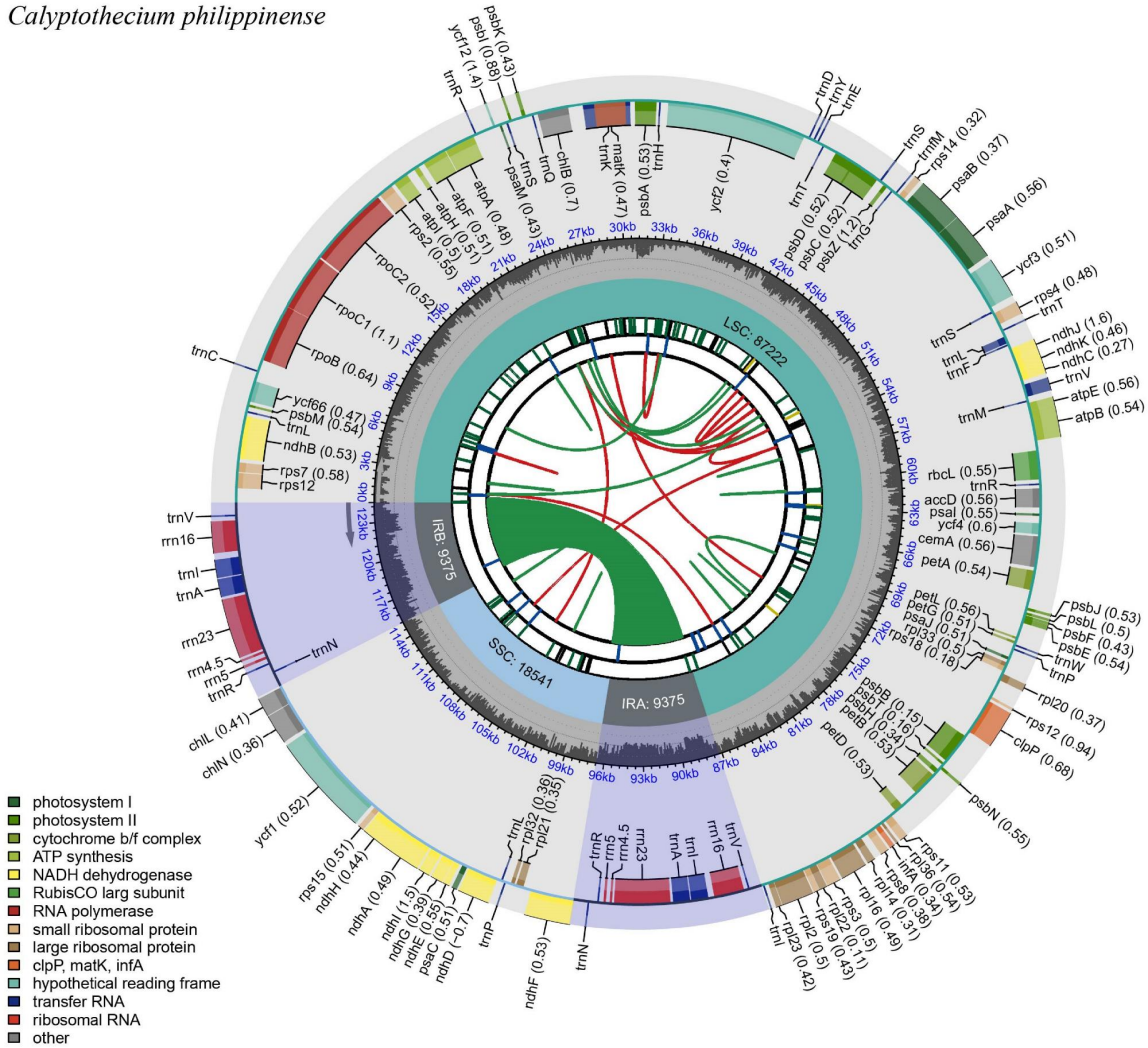
Gene map of the CPG of *C. philippinense* generated by CPGview (Liu et al. 2023). The map contains six tracks in default. From the center outward, the first track shows the dispersed repeats. The dispersed repeats consist of direct (D) and palindromic (P) repeats, connected with red and green arcs. The second track shows the long tandem repeats as short blue bars. The third track shows the short tandem repeats or microsatellite sequences as short bars with different colors. The small single-copy (SSC), inverted repeat (IRa and IRb), and large single-copy (LSC) regions are shown on the fourth track. The GC content along the genome is plotted on the fifth track. The genes are shown on the sixth track. The optional codon usage bias is displayed in the parenthesis after the gene name. Genes are color-coded by their functional classification. The transcription directions for the inner and outer genes are clockwise and anticlockwise, respectively. The functional classification of the genes is shown in the bottom left corner.

The length of the *C. philippinense* CPG was 124,513 bp (Figure 2, Figure S1), and the AT content was 74.2%. The quadripartite structure of the CPG comprised one small single-copy (SSC, 18,541 bp) region, one large single-copy (LSC, 87,222 bp) region, and two inverted repeat regions (IRA and IRB; each 9375 bp). The GC content of the SSC, LSC, and two inverted repeat (IRA and IRB) regions was 25.3%, 25.8%, and 44.9%, respectively. Cpgview (http://www.1kmpg.cn/cpgview/) detected 10 cis-splicing genes, including *ndh*B, *ycf*66, *rpo*C1, *atp*F, *ycf*3, *clp*P, *pet*B, *rpl*16, *rpl*2, and *ndh*A (Figure S2), and one trans-splicing genes *rps*12 (Figure S3). Although Cpgview failed to successfully generate the trans-splicing map file, we resorted to using PMGmap to create it (Figure S3). A total of 126 genes were annotated from the CPG of *C. philippinense*, including 82 protein-coding genes (PCGs), eight ribosomal RNA (rRNA) genes, and 36 transfer RNA (tRNA) genes. The genes could be divided into four categories according to their functions: (1) photosynthesis (47 genes); (2) regulation of expression (60 genes); (3) PCGs (six genes); and (4) unclear functions (five genes).

**Figure 3.**
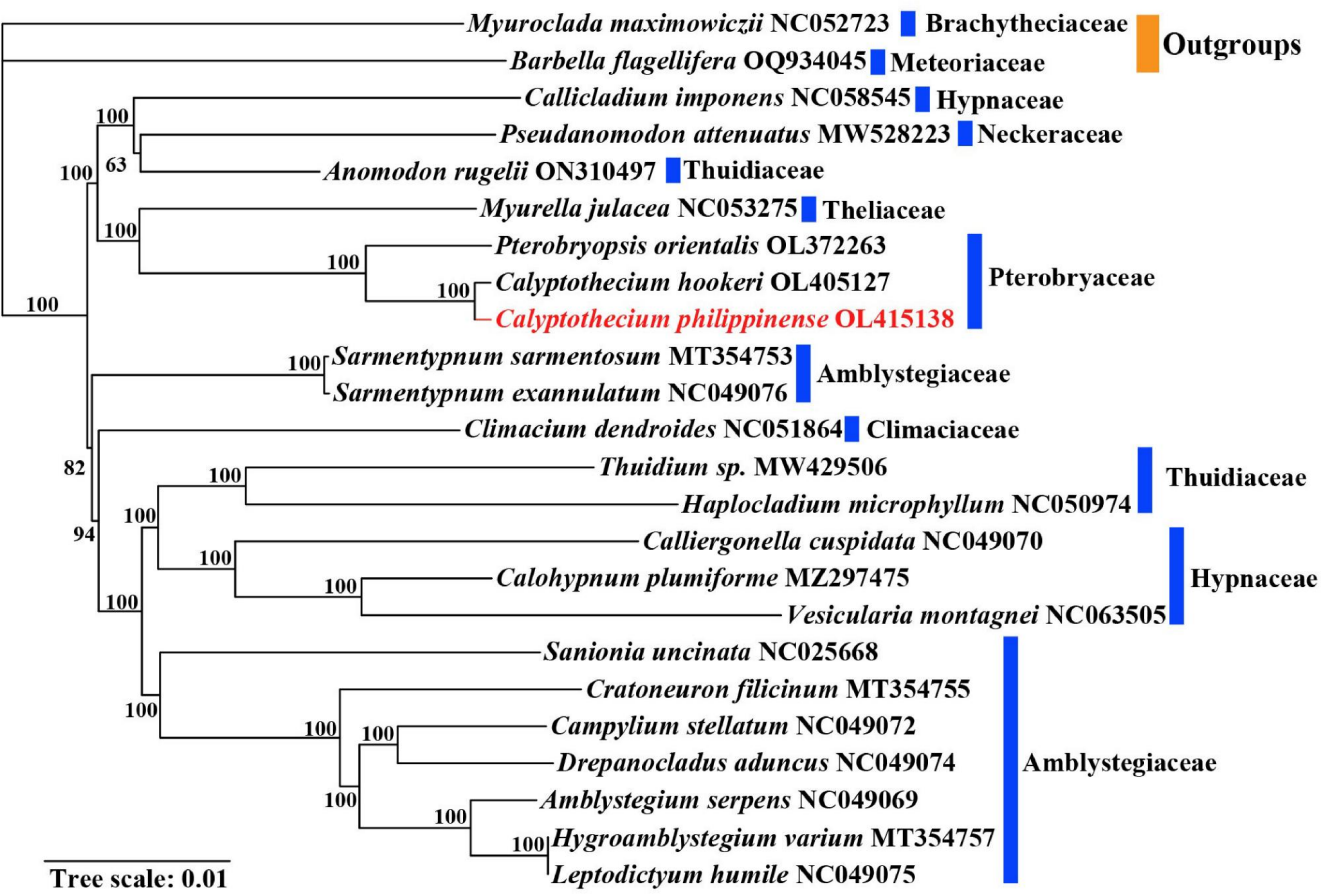
Maximum-likelihood (ML) phylogenetic tree based on the complete chloroplast genome sequence of 24 species from the Hypnales, and *Myuroclada maximowiczii* and *Barbella flagellifera* were selected as outgroups. GenBank accession numbers were listed behind each species name and the bootstrap support values are shown at the branches. The sequences used for constructing the phylogenetic tree are as follows: NC052723 (Han et al. 2020a), OQ934045 (Yuan et al. 2024), NC058545 (Asaf et al. 2024), MW528223 (Asaf et al. 2024), ON310497 (unpublished), NC053275 (Han et al. 2020c), OL372263 (Han et al. 2022b), OL405127 (Han et al. 2022a), OL415138 (the present study), MT354753 (Sheng et al. 2020), NC049076 (Sheng et al. 2020), NC051864 (Han et al. 2020b), MW429506 (Asaf et al. 2024), NC050974 (Mao et al. 2020), NC049070 (Sheng et al. 2020), MZ297475 (Ye et al. 2022), NC063505 (unpublished), NC025668 (Park et al. 2018), MT354755 (Sheng et al. 2020), NC049072 (Sheng et al. 2020), NC049074 (Sheng et al. 2020), NC049069 (Sheng et al. 2020), MT354757 (Sheng et al. 2020), NC049075 (Sheng et al. 2020).

Phylogenetic analysis of CPGs of 24 species in the order Hypnales showed that *C. philippinense* and *C. hookeri* formed a monophyletic clade, which formed as a sister clade to the species *P. orientalis* (Figure 3). We also found that within the Hypnales, the nine species of Amblystegiaceae, three species of Thuidiaceae, and four species of Hypnaceae failed to form a monophyletic group respectively. This phylogenetic result aligns closely with previous studies by Han et al. (2022a, 2022b), underscoring the consistent genetic architecture observed within Pterobryaceae.

## 4. Discussion and conclusion

Approximately 30 species have been described within the genus *Calyptothecium*, however, only one CPG has been deposited in the GenBank database so far (September 3, 2024). In this study, next-generation sequencing and assembly techniques were employed, and it was determined that the complete plastome of *Calyptothecium philippinense* is 124,513 bp in length. The structural characteristics of the chloroplast genome and its phylogenetic position within the Pterobryaceae were elucidated, and the gene order and composition were found to be consistent with those of typical plastomes of the Pterobryaceae, as reported by Han et al. (2022a, 2022b). Moreover, the phylogenetic tree based on the CPGs of 25 taxa in the Hypnales revealed that the three Pterobryaceae species—*C. philippinense*, *Calyptothecium hookeri*, and *Pterobryopsis orientalis*—formed a robust clade. In conclusion, this study not only offers significant insights for future taxonomic, systematic, and genetic research on Pterobryaceae, but also contributes to the understanding of genetic diversity and phylogenetic relationships within *Calyptothecium*.

## Supplementary Material

Fig S1.jpg

Fig S2.pdf

Fig S3.pdf

## Data Availability

The genome sequence data that obtained at this study are openly available in GenBank of NCBI (https://www.ncbi.nlm.nih.gov/) under accession no. OL415138. The associated BioProject, SRA, and BioSample numbers are PRJNA775849, SRR25302855, and SAMN24603894, respectively.

## References

[CIT0001] Asaf S, Jan R, Asif S, Bilal S, Khan AL, Kim K-M, Lee I-J, Al-Harrasi A. 2024. Plastome diversity and evolution in mosses: insights from structural characterization, comparative genomics, and phylogenetic analysis. Int J Biol Macromol, 257(Pt 2):128608. doi:10.1016/j.ijbiomac.2023.128608. 38065441

[CIT0002] Bartram EB. 1939. Mosses of the Philippines. Philipp J Sci. 68:234–238.

[CIT0003] Capella-Gutiérrez S, Silla-Martínez JM, Gabaldón T. 2009. trimAl: a tool for automated alignment trimming in large-scale phylogenetic analyses. Bioinformatics. 25(15):1972–1973. doi:10.1093/bioinformatics/btp348. 19505945 PMC2712344

[CIT0004] Dixon HN. 1937. Notulae Bryologicae I. J Bot Br Foreign. 75:121–129.

[CIT0005] Frey W, Stech M. 2009. Bryophyta (Musci, Mosses). In: Frey W, Stech M, Fischer E, editors. Syllabus of plant families: bryophytes and seedless vascular plants. Stuttgart (Germany): Borntraeger.

[CIT0006] Gangulee HC. 1976. Mosses of Eastern India and adjacent regions 5. Isobryales. Calcutta: Privately published; p. 1366–1382.

[CIT0007] Hall TA. 1999. BioEdit: a user-friendly biological sequence alignment editor and analysis program for Windows 95/98/NT. Nucleic Acids Symposium, 41; p. 95–98.

[CIT0008] Han W, Yu NN, Jia Y. 2022a. The complete chloroplast genome of *Calyptothecium hookeri* (Pterobryaceae, Hypnales). Mitochondrial DNA B Resour. 7(6):1046–1047. doi:10.1080/23802359.2022.2082893. 35756433 PMC9225731

[CIT0009] Han W, Yu NN, Li Y, Zhang L, Jia Y. 2022b. The complete chloroplast genome of *Pterobryopsis orientalis* (Pterobryaceae, Hypnales) and its phylogenetic implications. Mitochondrial DNA B Resour. 7(8):1484–1485. doi:10.1080/23802359.2022.2107448. 35989877 PMC9387310

[CIT0010] Han YD, Choi Y, Jang RH, Yoon YJ. 2020a. The complete chloroplast genome of a moss Korea *Myuroclada maximowiczii* (GG Borshch.) Steere & WB Schofield. Mitochondrial DNA Part B. 5(3):3443–3444. doi:10.1080/23802359.2020.1823265. 33458198 PMC7783053

[CIT0011] Han YD, Choi Y, Park S, Park YS, Yoon YJ. 2020b. The complete chloroplast genome of a moss Korea *Climacium dendroides* (Hedw.) F. Weber & D. Mohr. Mitochondrial DNA B Resour. 5(2):1200–1201. doi:10.1080/23802359.2020.1731362. 33366911 PMC7510824

[CIT0012] Han YD, Park S, Yoon YJ. 2020c. The complete chloroplast genome of the moss, *Myurella julacea* (Schwägr.) Schimp. (Bryidae, Pterigynandraceae). Mitochondrial DNA B Resour. 5(3):3505–3506. doi:10.1080/23802359.2020.1825138. 33458220 PMC7782937

[CIT0013] Hyvönen J. 1989. Bryophyte flora of the Huon Peninsula, Papua New Guinea. XXVI. Pterobryaceae (Musci). Acta Bot Fenn. 137:1–40.

[CIT0014] Jin J-J, Yu W-B, Yang J-B, Song Y, dePamphilis CW, Yi T-S, Li D-Z. 2020. GetOrganelle: a fast and versatile toolkit for accurate de novo assembly of organelle genomes. Genome Biol. 21(1):241–271. doi:10.1186/s13059-020-02154-5. 32912315 PMC7488116

[CIT0015] Kalyaanamoorthy S, Minh BQ, Wong TKF, von Haeseler A, Jermiin LS. 2017. ModelFinder: fast model selection for accurate phylogenetic estimates. Nat Methods. 14(6):587–589. doi:10.1038/nmeth.4285. 28481363 PMC5453245

[CIT0016] Kearse M, Moir R, Wilson A, Stones-Havas S, Cheung M, Sturrock S, Buxton S, Cooper A, Markowitz S, Duran C, et al. 2012. Geneious Basic: an integrated and extendable desktop software platform for the organization and analysis of sequence data. Bioinformatics. 28(12):1647–1649. doi:10.1093/bioinformatics/bts199. 22543367 PMC3371832

[CIT0017] Liu S, Ni Y, Li J, Zhang X, Yang H, Chen H, Liu C. 2023. CPGView: a package for visualizing detailed chloroplast genome structures. Mol Ecol Resour. 23(3):694–704. doi:10.1111/1755-0998.13729. 36587992

[CIT0018] Mao L, Ding H, Dong Q, Tian D. 2020. The chloroplast genome of the moss *Haplocladium microphyllum*, first in family Thuidiaceae. Mitochondrial DNA B Resour. 5(3):2813–2814. doi:10.1080/23802359.2020.1789006. 33457958 PMC7781994

[CIT0019] Nguyen LT, Schmidt HA, Haeseler AV, Minh BQ. 2015. IQ-TREE: a fast and effective stochastic algorithm for estimating maximum-likelihood phylogenies. Mol Biol Evol. 32(1):268–274. doi:10.1093/molbev/msu300. 25371430 PMC4271533

[CIT0020] Noguchi A. 1985. The Isobryalean mosses collected by Dr. Z. Iwatsuki in New Caledonia. J Hattori Bot Lab. 58:87–109.

[CIT0021] Park M, Park H, Lee H, Lee BH, Lee J. 2018. The complete plastome sequence of an Antarctic bryophyte *Sanionia uncinata* (Hedw.) Loeske. Int J Mol Sci. 19(3):709. doi:10.3390/ijms19030709. 29494552 PMC5877570

[CIT0022] Ronquist F, Teslenko M, van der Mark P, Ayres DL, Darling A, Höhna S, Larget B, Liu L, Suchard MA, Huelsenbeck JP. 2012. MrBayes 3.2: efficient Bayesian phylogenetic inference and model choice across a large model space. Syst Biol. 61(3):539–542. doi:10.1093/sysbio/sys029. 22357727 PMC3329765

[CIT0023] Sheng W, Yue XR, Li N, Liu Y, Wu YH. 2020. Comparison of eight complete plastid genomes from three moss families Amblystegiaceae, Calliergonaceae and Pylaisiaceae. Mitochondrial DNA B Resour. 5(3):3091–3093. doi:10.1080/23802359.2020.1797548. 33458070 PMC7782263

[CIT0024] Shi LC, Chen HM, Jiang M, Wang L, Wu X, Huang L, Liu C. 2019. CPGAVAS2, an integrated plastome sequence annotator and analyzer. Nucleic Acids Res. 47(W1):W65–W73. doi:10.1093/nar/gkz345. 31066451 PMC6602467

[CIT0025] Tan BC, Iwatsuki Z. 1991. A new annotated Philippine moss checklist. Harvard Pap Bot. 3:1–64.

[CIT0026] Tillich M, Lehwark P, Pellizzer T, Ulbricht-Jones ES, Fischer A, Bock R, Greiner S. 2017. GeSeq-versatile and accurate annotation of organelle genomes. Nucleic Acids Res. 45(W1):W6–W11. doi:10.1093/nar/gkx391. 28486635 PMC5570176

[CIT0027] Ye J, Ye J, Luo S, Chen J, Wen C, Xu Y. 2022. The complete chloroplast genome of *Calohypnum plumiforme* (Wilson) (Hypanceae, Bryophyta). Mitochondrial DNA B Resour. 7(3):480–481. doi:10.1080/23802359.2021.1996294. 35311207 PMC8928791

[CIT0028] Yu NN, Jia Y. 2015. A revision of the *Calyptothecium urvilleanum* complex (Pterobryaceae). J Bryol. 37(4):276–283. doi:10.1179/1743282015Y.0000000026.

[CIT0029] Yuan ZY, Wang Q, Sulayman M. 2024. Characterization of the complete chloroplast genome of *Barbella flagellifera* (Cardot) Nog. 1938 (Bryidae, Meteoriaceae). Mitochondrial DNA B Resour. 9(2):304–308. doi:10.1080/23802359.2024.2318393. 38414806 PMC10898263

[CIT0030] Zhang D, Gao F, Jakovlić I, Zou H, Zhang J, Li WX, Wang GT. 2020. PhyloSuite: an integrated and scalable desktop platform for streamlined molecular sequence data management and evolutionary phylogenetics studies. Mol Ecol Resour. 20(1):348–355. doi:10.1111/1755-0998.13096. 31599058

[CIT0031] Zhang X, Chen H, Ni Y, Wu B, Li J, Burzyński A, Liu C. 2024. Plant mitochondrial genome map (PMGmap): a software tool for the comprehensive visualization of coding, noncoding and genome features of plant mitochondrial genomes. Mol Ecol Resour. 24(5):e13952. doi:10.1111/1755-0998.13952. 38523350

