# Supplementary figures and images for: The complete chloroplast genome of Calyptothecium philippinense Broth. (Pterobryaceae, Hypnales)
Source: Mitochondrial DNA B Resour. 2024 Oct 7;9(10):1389–93. doi: 10.1080/23802359.2024.2412232 (PMC11459841; doi:10.1080/23802359.2024.2412232)

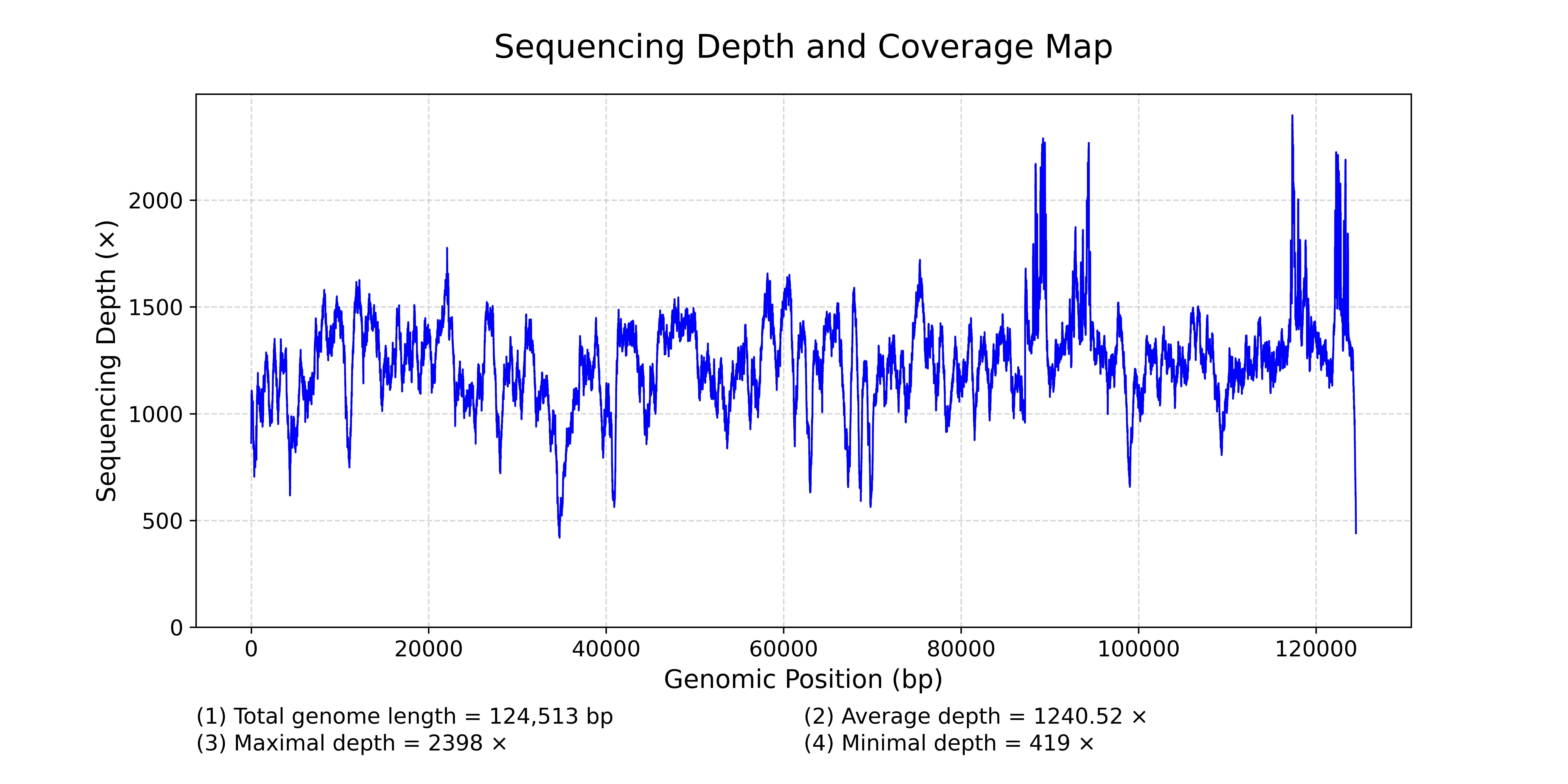

Supplement: Fig S1.jpg [file TMDN_A_2412232_SM1832.jpg]

# Cis-splicing Genes

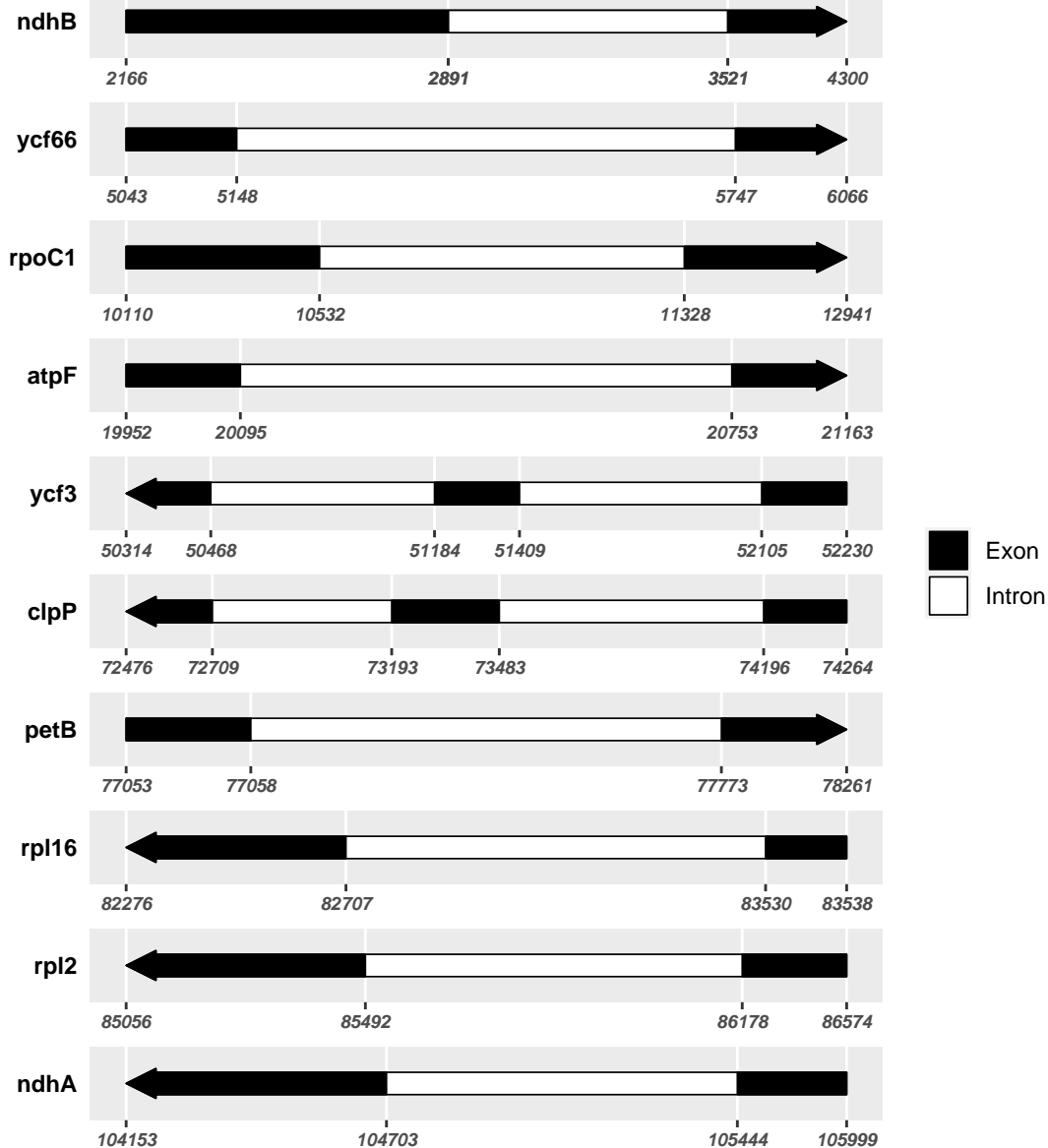

Supplement: Fig S2.pdf [file TMDN_A_2412232_SM1831.pdf]

# *Calyptothecium philippinense*

Plastid Genome  
trans-splicing genes

## *rps12*

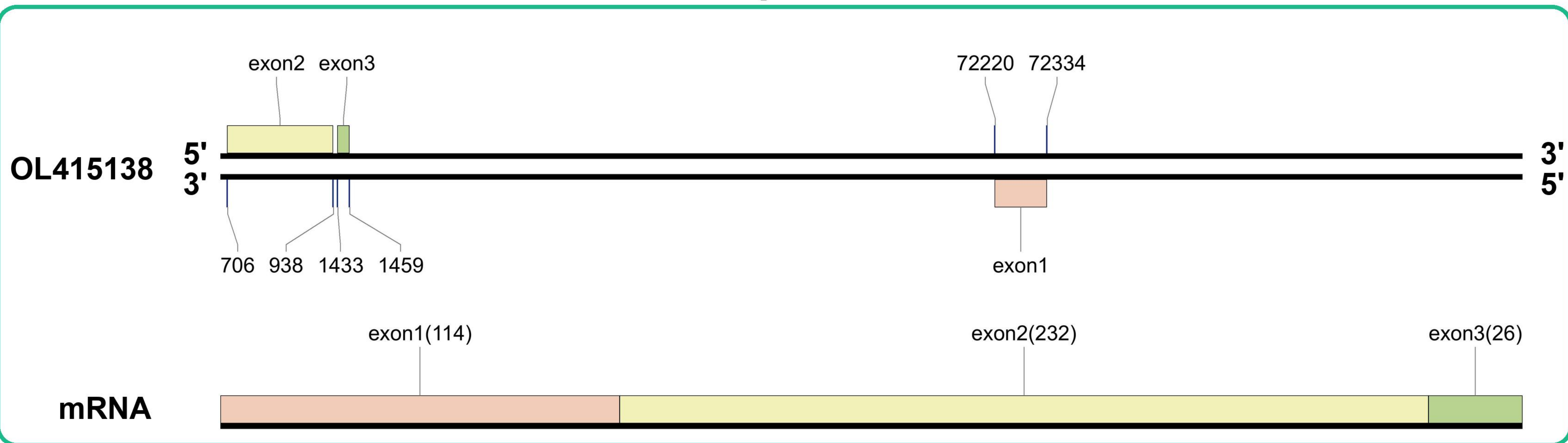

Supplement: Fig S3.pdf [file TMDN_A_2412232_SM1830.pdf]
